# Detection of SARS-CoV-2 variants in hospital wastewater in Peru, 2022

**DOI:** 10.17843/rpmesp.2024.412.13484

**Published:** 2024-05-14

**Authors:** Pool Marcos-Carbajal, José Yareta-Yareta, Miguel Otiniano-Trujillo, Marco Galarza-Pérez, Abraham Espinoza-Culupu, Jorge L. Ramirez-Melgar, Mario Chambi-Quispe, Néstor Alejandro Luque-Chipana, Rosmery Gutiérrez Ajalcriña, Victor Sucñer Cruz, Segundo Nicolas López Chegne, Diana Santillán Ruiz, Luis Felipe Segura Chavez, Cinthia Esther Sias Garay, Alberto Salazar Granara, Pablo Tsukayama Cisneros, Silvana Teresa Tapia Paniagua, Carmen María González-Domenech

**Affiliations:** 1 Universidad Peruana Unión, Professional School of Medicine, Research Laboratory in Molecular Biology. Lima Peru. Universidad Peruana Unión Universidad Peruana Unión Professional School of Medicine Research Laboratory in Molecular Biology Lima Peru; 2 Instituto Nacional de Salud, National Center for Public Health, Laboratory of Biotechnology and Molecular Biology. Lima Peru. Instituto Nacional de Salud National Center for Public Health Laboratory of Biotechnology and Molecular Biology Lima Peru; 3 Universidad Nacional Mayor de San Marcos, Faculty of Biological Sciences. Lima, Peru. Universidad Nacional Mayor de San Marcos Universidad Nacional Mayor de San Marcos Faculty of Biological Sciences Lima Peru; 4 Carlos Monge Medrano Hospital, Clinical Pathology. Puno, Peru. Carlos Monge Medrano Hospital Clinical Pathology Puno Perú; 5 Ate Vitarte Hospital, Intensive Care Unit. Lima Peru. Ate Vitarte Hospital Intensive Care Unit Lima Peru; 6 Huaycan Hospital, Epidemiology Area. Lima Peru. Huaycan Hospital Epidemiology Area Lima Peru; 7 Regional Hospital of Cusco, Clinical Pathology. Cusco, Peru. Regional Hospital of Cusco Clinical Pathology Cusco Peru; 8 Regional Hospital of Cajamarca, Clinical Pathology. Cajamarca, Peru. Regional Hospital of Cajamarca Clinical Pathology Cajamarca Peru; 9 Tarapoto Hospital, Department of Pathological Anatomy and Clinical Pathology. Tarapoto, Peru. Tarapoto Hospital Department of Pathological Anatomy and Clinical Pathology Tarapoto Peru; 10 San Martin de Porres University, Center for Research in Traditional Medicine and Pharmacology. Lima Peru. San Martin de Porres University San Martin de Porres University Center for Research in Traditional Medicine and Pharmacology Lima Peru; 11 Universidad Peruana Cayetano Heredia, Laboratory of Microbial Genomics. Lima Peru. Universidad Peruana Cayetano Heredia Universidad Peruana Cayetano Heredia Laboratory of Microbial Genomics Lima Peru; 12 University of Malaga, Department of Microbiology, Faculty of Sciences. Malaga, Spain. University of Malaga University of Malaga Department of Microbiology Faculty of Sciences Málaga Spain

**Keywords:** Genomics, SARS-CoV-2, Wastewater, Sequencing, Wastewater-Based Epidemiological Monitoring

## Abstract

**Objective.:**

To identify the presence of the SARS-CoV-2 virus in wastewater from hospitals in Peru.

**Materials and methods.:**

Water samples were collected from the effluents of nine hospitals in Peru during March and September 2022. SARS-CoV-2 was identified by using Illumina sequencing. Variant, lineage and clade assignments were carried out using the Illumina and Nextclado tools. We verified whether the SARS-CoV-2 variants obtained from wastewater were similar to those reported by the National Institute of Health of Peru from patients during the same period and region.

**Results.:**

Eighteen of the 20 hospital wastewater samples (90%) provided sequences of sufficient quality to be classified as the Omicron variant according to the WHO classification. Among them, six (30%) were assigned by Nextclade to clades 21K lineage BA.1.1 (n=1), 21L lineage BA.2 (n=2), and 22B lineages BA.5.1 (n=2) and BA .5.5 (n=1).

**Conclusions.:**

SARS-CoV-2 variants were found in hospital wastewater samples and were similar to those reported by the surveillance system in patients during the same weeks and geographic areas. Wastewater monitoring could provide information on the environmental and temporal variation of viruses such as SARS-CoV-2.

## INTRODUCTION

RNA viruses, such as Ebola, influenza, dengue, Zika, and SARS-CoV-2, evolved rapidly, steadily accumulating mutations in their genomes ^1^^,^^2^. This feature can be used to make epidemiological inferences and identify risk factors associated with transmission events occurring in susceptible populations ^1^^,^^3^. Since December 2019, a large number of SARS-CoV-2 genomes have been generated worldwide and deposited in public repositories (i.e., GISAID, Genbank), allowing us to track, almost in real time, the evolution of this virus ^2^^-^^5^. However, we only found studies on transmission patterns in their local populations in a few European and North American countries ^6^^,^^7^.

Latin America has generated more than 100,000 genomes, mainly from Brazil, Chile, Peru, Colombia, Ecuador, and Uruguay ^2^^,^^5^. For example, the Peruvian Institute of Health (INS) in collaboration with the National Center for Epidemiology, Prevention and Disease Control of the Ministry of Health (CDC-MINSA) in Peru, have generated more than 54,506 SARS-CoV-2 genomes by 2024 (https://gisaid.org/) confirming the circulation of a myriad of variants since the onset of the COVID-19 pandemic (i.e., lambda, gamma, alpha, delta, mu, zeta, epsilon, etc. and others (https://nextstrain.org/ncov/gisaid/21L/south-america/1m) ^8^^-^^11^.

Complementary to clinical detection, environmental surveillance of viruses, especially those related to viability and potential infectivity such as SARS-CoV-2, could be a useful tool to predict timely disease outbreaks and issue early warnings by health authorities ^12^^,^^13^. In that sense, wastewater samples are a noninvasive and inexpensive source of information to investigate the spread of different genetic variants of SARS-CoV-2 within a community ^14^^,^^15^. Mass sequencing and metagenomic analysis would allow us to detect the virus and identify circulating SARS CoV-2 variants at the same time.

In this study, we aimed to detect, by next-generation sequencing, SARS-CoV-2 variants from different hospital effluents in Peru during March and September 2022. The recovered data were matched with circulating variants monitored by the Peruvian INS surveillance system.

**KEY MESSAGES**

Motivation for the study: To contribute to the surveillance of environmental samples from hospital effluents in order to achieve early warning of possible infectious disease outbreaks.

Main findings:The Omicron variant of the COVID-19 virus was detected in wastewater from hospitals in Puno, Cuzco and Cajamarca; these results are similar to the reports by the Peruvian National Institute of Health based on nasopharyngeal swab samples .

Implications: The presence of the Omicron variant in hospital wastewater during the third wave of the pandemic should raise awareness of the treatment system before wastewater is discharged into the public sewer system.

## MATERIALS AND METHODS

### Procedures

**Figure 1:**
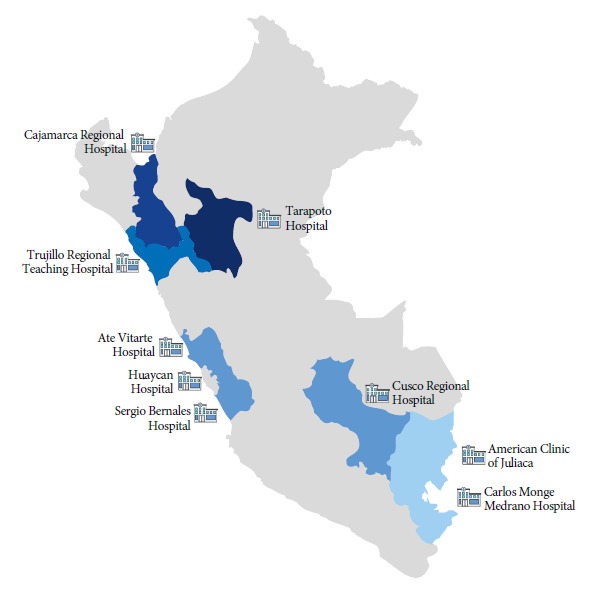
Hospitals by region sampled between March and September 2022 in Peru.

Samples were collected from wastewater of nine hospitals located in six regions of Peru (Lima=3, San Martin=1, Puno=2, Cusco=1, Cajamarca=1, La Libertad=1) between March and September 2022 (Figure 1). Each hospital was geo-referenced and two samples were collected at each point during a one-hour period. Wastewater samples were passively collected in sterile glass bottles (1000 mL) and then labeled. Samples were transported under cold chain conditions at 8 °C and processed within 24 hours. Once the extraction of nucleic acids was completed, they were stored at -20°C in the molecular biology laboratory of the Universidad Peruana Unión, following the recommendations by the U.S. CDC (https://www.cdc.gov/nwss/wastewater-surveillance.html).

### Sample preparation and pretreatment

We followed the protocol of the Wizard® Enviro Wastewater FB236 total nucleic acid kit (Promega Corp, USA). Briefly, a total of 40 mL of homogenized wastewater samples were treated with 500 μL of proteinase K, mixed by inversion and incubated for 30 min at room temperature, then centrifuged at 3000 g for 10 min, following the manufacturer’s instructions. Subsequently, we added 6 mL of binding buffer 1 and then 0.5 mL of binding buffer 2 (both included in the same kit) and mixed by inversion. Finally, 24 mL of isopropanol (Sigma Aldrich Co., St. Louis, MO, USA) was added to each tube. The mixture was passed through the PureYieldTM Minicolumn according to the same protocol. Finally, the columns were subjected to a vacuum system, and the total content was finally eluted with 1000 μL of preheated RNase/DNase-free water (60 °C).

### RNA purification and electrophoresis analysis

The eluate obtained in the previous step was purified to obtain total RNA using silicaPureYield^TM^ mini-columns, also provided by Wizard® Enviro Wastewater FB236 Total Nucleic Acid Kit (Promega Corp, USA), following the manufacturer’s instructions. The total eluate content was run through the entire column, washed and finally recovered with 70 μL of RNase/DNase-free water, at 11000 g for one minute, following the manufacturer’s instructions. RNAs were quantified by absorbance at 260 nm using a Nanodrop ^16^^)^ and integrity was visualized on 1% agarose gels (Sigma Aldrich Co, St. Louis, MO, USA) stained with Sybr gold.

### cDNA and library preparation and NGS

Total RNA was retrotranscribed to cDNA using random primers, and the library was prepared using the Illumina COVID Seq Kit RUO (Illumina San Diego, California, USA). The MiSeq kit (Illumina®) was used for sequencing according to the manufacturer’s instructions. Sequencing was outsourced and performed by Genlab del Perú S.A.C.

### Bioinformatic analysis

The quality of the readings was evaluated using software implemented in the Illumina® tool package, with additional analysis performed by Nextclade and Pangolin software ^17^^,^^18^. These two programs allowed us to identify which variants and lineages were circulating in Peru. Additionally, the findings (variants and lineages) were compared with those from clinical samples tracked by the INS of Peru during the same period and geographic area (https://web.ins.gob.pe/es/covid19/secuenciamiento-sars-cov2).

## RESULTS

Sequences were obtained from each sample, gathering 20 sequences in total. However, we initially assessed their quality with the Pangolin and Nextclade programs, and all were discarded by the first program as they were labeled as ambiguous content or failure, but the second program retrieved six results. As shown, all sequences with sufficient quality were assigned to the Omicron variant according to the World Health Organization (WHO) label. However, the lineages were diverse within the three clades found: the 21K clade with the BA.1.1 lineage (n=1); the 21L clade with the BA.2 lineage (n=2); and the 22B clade with the BA.5.1 (n=2) and BA.5.5 (n=1) lineages (Table 1).

**Table 1:** SARS-CoV-2 lineage sequences from wastewater samples in nine hospitals in Peru, 2022.

Hospital (Region)	Sample No.	Georeferentiation		Clinical surveillance data		Wastewater sample		
		Latitude	Longitude	Epidemiological week *	Variants and predominant lineages of SARS-CoV-2 (WHO)*	SARS-CoV-2 variant (assignment of the following clade)	Linage	Clade
Carlos Monge Medrano Hospital (Puno)	1	-15.48103444	-70.12079352	24	Omicron, Linages BA.2.12.1, BA.4.1, BA.5.1, BA.5.2, BA.4.6, BA.5.1.8	NA		
	2			35	Omicron, Linages BA.5, BA.5.1, BA.5.2.1, BA.5.2, BA.5.6	Omicron	BA.5.1	22B
Regional Teaching Hospital of Trujillo (La Libertad)	3	-8.105569975	-79.03658615	24	Omicron, Linages BA.2, BA.2.5, BA.4, BA.5, BA.2.12.1, BA.4.1, BA.5.1, BA.2.9	NA		
	4			28	Omicron, Linages BA.2, BA.4, BA.2.12.1, BA.4.1, BA.5.1, BA.5.2, BA.4.6	NA		
Ate Vitarte Emergency Hospital (Lima)	5	-12.02584008	-76.91729777	25	Omicron, Linages BA.2, BA.4, BA.5, BA.2.12.1	NA		
	6					NA		
	7					NA		
Huaycan Hospital (Lima)	8	-12.01544188	-76.82024846	19	Omicron, Linages BA.1, BA.1.1, BA.2, BA.4, BA.5, BA.2.12.1	NA		
	9					NA		
Cusco Regional Hospital (Cusco)	10	-13.52354454	-71.95475727	32	Omicron, Linages BA.2, BA.4, BA.5, BA.5.2, BA.2.12.1, BA.4.1, BA.5.1, BA.5.1.1, BA.5.2.1, BE.1, BA.4.6, BA.5.6, BA.5.6.1	Omicron	BA.2	21L
	11					Omicron	BA.2	21L
Juliaca American Clinic (Puno)	12	-15.49743882	-70.13242485	35	Omicron, Linages BA.5, BA.5.1, BA.5.2.1, BA.5.2, BA.5.6	Omicron	BA.5.5	22B
	13					Omicron	BA.5.1	22B
Cajamarca Regional Hospital (Cajamarca)	14	-7.183099124	-78.48777246	34	Omicron, Linages BA.4, BA.5, BA.4.1, BA.5.1, BA.5.2.1, BA.5.2, BA.4.6, BA.5.1.3, BA.5.6, BA.5.1.3, BA.5.6.1	Omicron	BA.1.1	21K
	15					NA		
Sergio Bernales Hospital (Lima)	16	-11.91352635	-77.03915675	10	Omicron, Linages BA.1, BA.1.1, BA.2	NA		
	17					NA		
Tarapoto Hospital (San Martín)	18	-6.473262643	-6.473262643	34	Omicron, Linages BA.4.1, BA.5.1, BA.5.1.1, BA.5.2.1, BA.5.6.1	NA		
	19					NA		
	20					NA		

* Assignment according to the National Institute of Health of Peru for the location and week of sampling. It was not possible to recover the data for Puno during week 24 (06/16/2022), so the data shown is from July 2022.

When we compared our results with the samples obtained from patients monitored by the INS during the same period, we found a similar presence in both the variant, all were Omicron, the same variant circulating in the Peruvian population, and the lineages. Only BA.5.5, was not reported by INS for the area and time of sampling in the Puno region (Table 1).

All 14 sequences, except one (WW20) discarded by Nextclade software due to its low quality, were analyzed by Illumina tools and appeared to belong to Omicron clade 19A. However, this result could not be considered robust (the other tools had discarded them), but somehow it also showed the underlying relationship with the Omicron variant.

## DISCUSSION

The COVID-19 pandemic in Peru was characterized by a high number of cases, deaths per million population, and total excess deaths compared with other South American countries ^19^. Several causes have been suggested to explain these figures, but they may have been caused by a leaderless and uncoordinated public health strategy, as well as to the lack of infrastructure ^20^^,^^21^. Paradoxically, Peru was the first country in Latin America to impose strict containment in 2020, achieving a rapid improvement of the first wave of cases in June 2020 ^22^. However, the relaxation of restrictions in August 2020 was associated with a second wave later that year ^21^. The new peak of cases between December 2020 and February 2021 again forced restriction measures and targeted quarantines in areas with the highest incidence of cases. One year later, the Peruvian Ministry of Health announced the third wave, which started in January 2022 and lasted up to April 2022 ^23^^,^^24^. This study took place in the context of this third wave and aimed to detect SARS-CoV-2 variants from different hospital effluents in Peru during the period from March to September 2022 and covered health facilities on the coast, jungle, and highland regions, showing genetic material found in the analyzed samples, which would indicate that the aforementioned lineages would be circulating in that time period.

SARS-CoV-2 variants have been detected from respiratory samples in Peru, some of them are variants of interest (VOI) and other variants of concern (VOC), according to the CDC and WHO classification based on their impact on public health ^25^^,^^26^. Thus, the Lambda and Gamma lineages circulated during the second wave of the pandemic in Peru. Lambda focused on the coastal and highland regions, while Gamma focused on the jungle region (Loreto) ^11^^,^^27^. In fact, the Lambda variant (C.37) was detected worldwide for the first time in Peru in August 2020. On the other hand, the third wave was dominated by lineages descended from Omicron BA.1 (B.1.1.529); this variant was the only one found in our study. In addition, the descendant lineages found in the wastewater samples matched some of those detected by INS in patients from the same geographic area and week of sampling. As previously reported by other studies, detection of SARS-CoV-2 in wastewater may reflect the spread of this virus in the community ^28^^,^^29^. In addition, this approach allows us to observe undetected transmission of SARS-CoV-2 variants ^30^. In that regard, we found an Omicron lineage, BA.5.5, which was not reported by INS for the sampling locations and period, but could have been circulating.

The main limitation of our study was the quality of the samples, which forced us to discard a considerable number of samples. Low-quality sequences, in addition to the difficulty in estimating relative lineage abundance in complex samples, are not uncommon when conducting wastewater surveillance ^31^. The low prevalence of different lineages in these samples is probably the main reason ^32^, which would also explain our results. Besides, the timing of sampling did not coincide with the large peak of cases reported during the third wave and where many lineages were detected in the population.

The strength of the study lies in the fact that it is one of the first studies to report SARS-CoV-2 variants in hospital wastewater in Peru. Genomic surveillance based on sequencing provides us with this important information to learn more about the ongoing transmission of the virus in the community. This study involved different researchers who are part of the antimicrobial resistance network and who have contributed their experience and knowledge to achieve the proposed objectives; the support of different actors and institutions was necessary. Despite the limitations, our study is another example of how wastewater sequencing analysis could be useful as a counterpart for viral surveillance in patients, especially in countries where clinical testing is still a challenge.

In conclusion, the presence of SARS-CoV-2 variants in hospital wastewater samples is evident and were similar to those reported by the surveillance system in patients during the same weeks and geographic areas in Peru. Wastewater monitoring contributes to provide information on the environmental and temporal variation of viruses such as SARS-CoV-2.
