# Supplementary material for: Detection of SARS-CoV-2 variants in hospital wastewater in Peru, 2022
Source: Rev Peru Med Exp Salud Publica. 2024 May 14;41(2):140–5. doi: 10.17843/rpmesp.2024.412.13484 (PMC11300693; doi:10.17843/rpmesp.2024.412.13484)
Supplement: Translation

# Translation: Detección de variantes del SARS-CoV-2 en aguas residuales de hospitales en Perú, 2022

**Authors:** Pool Marcos-Carbajal, José Yareta-Yareta, Miguel Otiniano-Trujillo, Marco Galarza-Pérez, Abraham Espinoza-Culupu, Jorge L. Ramirez-Melgar, Mario Chambi-Quispe, Néstor Alejandro Luque-Chipana, Rosmery Gutiérrez Ajalcriña, Victor Sucñer Cruz, Segundo Nicolas López Chegne, Diana Santillán Ruiz, Luis Felipe Segura Chavez, Cinthia Esther Sias Garay, Alberto Salazar Granara, Pablo Tsukayama Cisneros, Silvana Teresa Tapia Paniagua, Carmen María González-Domenech

**Affiliations:** 1 Universidad Peruana Unión, Escuela Profesional Medicina, Laboratorio de Investigación en Biología Molecular. Lima, Perú.; 2 Instituto Nacional de Salud, Centro Nacional de Salud Pública, Laboratorio de Biotecnología y Biología molecular. Lima, Perú.; 3 Universidad Nacional Mayor de San Marcos, Facultad de Ciencias Biológicas. Lima, Perú.; 4 Hospital Carlos Monge Medrano, Patología Clínica. Puno, Perú.; 5 Hospital de Ate Vitarte, Unidad de Cuidados Intensivos. Lima, Perú.; 6 Hospital de Huaycan, Área de Epidemiología. Lima, Perú.; 7 Hospital Regional del Cusco, Patología Clínica. Cusco, Perú.; 8 Hospital Regional de Cajamarca, Patología Clínica. Cajamarca, Perú.; 9 Hospital de Tarapoto, Departamento de Anatomía Patológica y Patología Clínica. Tarapoto, Perú.; 10 Universidad San Martin de Porres, Centro de Investigación en Medicina Tradicional y Farmacología. Lima, Perú.; 11 Universidad Peruana Cayetano Heredia, Laboratorio de Genómica Microbiana. Lima, Perú.; 12 Universidad de Málaga, Departamento de Microbiología, Facultad de Ciencias. Málaga, España.

**Keywords:** Genómica, SARS-CoV-2, Aguas residuales, Secuenciación, Monitoreo epidemiológico basado en aguas residuales

## Abstract

**Objetivo.:**

Identificar la presencia del virus SARS-CoV-2 en aguas residuales de hospitales en Perú.

**Materiales y métodos:**

. Se recolectaron muestras de agua en los efluentes de nueve hospitales del Perú durante marzo y septiembre de 2022 y se realizó la identificación de SARS-CoV-2 mediante secuenciación Illumina. Las asignaciones de variantes, linajes y clados se llevaron a cabo con las herramientas Illumina y Nextclado. Verificamos si las variantes de SARS-CoV-2 obtenida de las aguas residuales fueron similares a las reportadas por el Instituto Nacional de Salud del Perú procedentes de pacientes durante el mismo período y región.

**Resultados:**

. Dieciocho de las 20 muestras de aguas residuales hospitalarias (90%) proporcionaron secuencias con la calidad suficiente para ser clasificadas como variante Ómicron según la clasificación de la OMS. Entre ellos, seis (30%) fueron asignados por Nextclade a los clados 21K linaje BA.1.1 (n=1), 21 L linaje BA.2 (n=2) y 22B linajes BA.5.1 (n=2) y BA.5.5 (n=1).

**Conclusiones:**

. Se encontraron variantes del SARS-CoV-2 en muestras de aguas residuales hospitalarias y que fueron similares a las reportadas por el sistema de vigilancia en pacientes durante las mismas semanas y áreas geográficas. El monitoreo de aguas residuales podría proporcionar información sobre la variación ambiental y temporal de virus como el SARS-CoV-2.

## INTRODUCCIÓN

Los virus de ARN, como el ébola, la gripe, el dengue, el zika y el SARS-CoV-2, evolucionaron rápidamente con una acumulación constante de mutaciones en sus genomas ^1^^,^^2^. Esta característica puede utilizarse para hacer inferencias epidemiológicas e identificar los factores de riesgo asociados a los eventos de transmisión que ocurren en poblaciones susceptibles ^1^^,^^3^. Desde diciembre de 2019, se ha generado una gran cantidad de genomas de SARS-CoV-2 en todo el mundo y se han depositado en repositorios públicos (i.e., GISAID, Genbank), lo que nos permite rastrear, casi en tiempo real, la evolución de este virus ^2^^-^^5^. Sin embargo, solo encontramos estudios sobre los patrones de transmisión en sus poblaciones locales de unos pocos países europeos y norteamericanos ^6^^,^^7^.

Latinoamérica ha generado más de 100 000 genomas, especialmente de Brasil, Chile, Perú, Colombia, Ecuador y Uruguay ^2^^,^^5^. Por ejemplo, el Instituto Nacional de Salud (INS) en colaboración con el Centro Nacional de Epidemiología, Prevención y Control de Enfermedades del Ministerio de Salud (CDC-MINSA) en el Perú, han generado más de 54506 genomas de SARS-CoV-2 para el 2024 (https://gisaid.org/) confirmando la circulación de una miríada de variantes desde el inicio de la pandemia de COVID-19 (i.e., lambda, gamma, alfa, delta, mu, zeta, épsilon, etc. y otros. (https://nextstrain.org/ncov/gisaid/21L/south-america/1m) ^8^^-^^11^.

De forma complementaria a la detección clínica, la vigilancia ambiental de los virus, especialmente los relacionados con la viabilidad y la potencial infectividad como el SARS-CoV-2, podría actuar como una herramienta útil para predecir brotes oportunos de enfermedades y emitir una alerta rápida por parte de las autoridades sanitarias ^12^^,^^13^. En ese sentido, las muestras de aguas residuales son una fuente de información no invasiva y económica para investigar la propagación de diferentes variantes genéticas del SARS-CoV-2 dentro de una comunidad ^14^^,^^15^. La secuenciación masiva y el análisis metagenómico nos permitirían detectar el virus e identificar las variantes circulantes del SARS CoV-2 al mismo tiempo.

En este estudio, nos propusimos detectar mediante secuenciación de nueva generación las variantes de SARS-CoV-2 provenientes de diferentes efluentes hospitalarios en Perú, durante marzo y septiembre de 2022. Los datos recuperados fueron cotejados con las variantes circulantes monitoreadas por el sistema de vigilancia del INS del Perú.

**MENSAJE CLAVE**

Motivación para realizar el estudio: Contribuir a la vigilancia de las muestras ambientales de procedencia hospitalaria para emitir alertas tempranas ante posibles brotes de enfermedades infecciosas.

Principales hallazgos: La variante Ómicron del virus de la COVID-19 se detectó en aguas residuales de hospitales en Puno, Cuzco y en Cajamarca, esta variante es similar al reportado por Instituto Nacional de Salud del Perú en base a muestras de hisopos nasofaríngeos.

Implicancias: La presencia de la variante Ómicron en las aguas residuales hospitalarias durante la tercera ola de la pandemia debe generar conciencia sobre el sistema de tratamiento antes de que las aguas residuales se viertan en el sistema de alcantarillado público.

## MATERIALES Y MÉTODOS

### Procedimientos

Las muestras fueron recolectadas en colectores de aguas residuales de nueve hospitales ubicados en seis regiones del Perú (Lima=3, San Martín=1, Puno=2, Cusco=1, Cajamarca=1, La Libertad=1) entre marzo y septiembre de 2022 (Figura 1). Cada hospital fue georreferenciado y en cada punto se tomaron dos muestras en un período de una hora. Las muestras de agua residual se recolectaron pasivamente en botellas de vidrio estériles (1000 mL) y luego se etiquetaron. Las muestras se transportaron en condiciones de cadena de frío a 8 °C y se procesaron en un plazo de 24 horas. Una vez finalizada la extracción de ácidos nucleicos, estos fueron almacenados a -20°C en el laboratorio de biología molecular de la Universidad Peruana Unión, siguiendo las recomendaciones del CDC de EE. UU. (https://www.cdc.gov/nwss/wastewater-surveillance.html).

**Figura 1:** Hospitales según región muestreados entre marzo y septiembre de 2022 en Perú

### Preparación y pretratamiento de las muestras

Seguimos el protocolo del kit de ácido nucleico total FB236 de Wizard® Enviro Wastewater (Promega Corp, EE. UU.). Brevemente, un total de 40 mL de muestras de aguas residuales homogeneizadas fueron tratadas con 500 μL de proteinasa K, mezcladas por inversión e incubadas durante 30 minutos a temperatura ambiente, luego centrifugadas a 3000 g durante 10 minutos, siguiendo las instrucciones del fabricante. Posteriormente, añadimos 6 mL de tampón de unión 1 y luego 0,5 mL de tampón de unión 2 (ambos incluidos en el mismo kit) y mezclamos bien por inversión. Finalmente, se añadieron 24 mL de isopropanol (Sigma Aldrich Co., St. Louis, MO, Estados Unidos) a cada tubo. La mezcla se pasó a través de la columna PureYield^TM^ Minicolumn, de acuerdo con el mismo protocolo. Para finalizar, las columnas se sometieron a un sistema de vacío, y el contenido total finalmente se eluyó con 1000 μL de agua libre de RNasa/DNasa precalentada (60 °C).

### Purificación de ARN y análisis de electroforesis

El eluido obtenido en el paso anterior se purificó para obtener ARN total utilizando las minicolumnas de sílicePureYield ^TM^ , también proporcionadas por el kit de ácido nucleico total FB236 de Wizard® Enviro Wastewater (Promega Corp, EE. UU.), siguiendo las instrucciones del fabricante. El contenido total del eluido se pasó por toda la columna, se lavó y finalmente se recuperó con 70 μL de agua libre de RNasa/DNasa, a 11000 g durante un minuto, siguiendo las instrucciones del fabricante. Los ARN se cuantificaron por absorbancia a 260 nm utilizando un Nanodrop ^16^ y la integridad se visualizó en geles de agarosa al 1% (Sigma Aldrich Co, St. Louis, MO, EE. UU.) teñidos con oro Sybr. 

### ADNc y preparación de bibliotecas y NGS

El ARN total se retrotranscribe a ADNc utilizando cebadores aleatorios, y la preparación de la biblioteca se realizó utilizando el Illumina COVID Seq Kit RUO (Illumina San Diego, California, EE. UU.). Se utilizó el equipo MiSeq (Illumina®) para secuenciar de acuerdo con las instrucciones del fabricante. La secuenciación fue tercerizada y realizada por Genlab del Perú S.A.C.

### Análisis bioinformático

La calidad de las lecturas se evaluó mediante un software implementado en el paquete de herramientas Illumina®, con un análisis adicional realizado por los softwares Nextclade y Pangolin ^17^^,^^18^. Estos dos programas nos permitieron identificar qué variantes y linajes estaban circulando en el Perú. Adicionalmente, se evaluaron los hallazgos (variantes y linajes) con los de muestras clínicas rastreadas por el INS del Perú durante el mismo periodo y área geográfica (https://web.ins.gob.pe/es/covid19/secuenciamiento-sars-cov2).

## RESULTADOS

Se obtuvieron secuencias de cada muestra, reuniendo 20 secuencias en total. Sin embargo, los programas Pangolin y Nextclade evaluaron primero su calidad, siendo descartados todos por el primer programa al ser etiquetados como contenido ambiguo o falla, y fueron recuperados seis resultados por el segundo programa. Como se muestra, todas las secuencias con calidad suficiente se asignaron a la variante Ómicron de acuerdo con la etiqueta de la Organización Mundial de la Salud (OMS). Sin embargo, los linajes fueron diversos dentro de los tres clados encontrados: el clado 21K con el linaje BA.1.1 (n=1); el clado 21L con el linaje BA.2 (n=2); y el clado 22B con los linajes BA.5.1 (n=2) y BA.5.5 (n=1) (Tabla 1).

**Tabla 1:** Secuencias de linajes SARS-CoV-2 a partir de muestras de aguas residuales en nueve hospitales de Perú 2022.

Hospital (Región)	Nro. de muestra	Georreferenciación		Datos de vigilancia clínica		Muestra de aguas residuales		
		Latitud	Longitud	Semana epidemiológica*	Variantes y linajes predominantes del SARS-CoV-2 (OMS)*	Variante del SARS-CoV-2 (asignación del siguiente clado)	Linaje	Clado
Hospital Carlos Monge Medrano (Puno)	1	-15.48103444	-70.12079352	24	Ómicron, Linajes BA.2.12.1, BA.4.1, BA.5.1, BA.5.2, BA.4.6, BA.5.1.8	NA		
	2			35	Ómicron, Linajes BA.5, BA.5.1, BA.5.2.1, BA.5.2, BA.5.6	Ómicron	BA.5.1	22B
Hospital Regional Docente de Trujillo (La Libertad)	3	-8.105569975	-79.03658615	24	Ómicron, Linajes BA.2, BA.2.5, BA.4, BA.5, BA.2.12.1, BA.4.1, BA.5.1, BA.2.9	NA		
	4			28	Ómicron, Linajes BA.2, BA.4, BA.2.12.1, BA.4.1, BA.5.1, BA.5.2, BA.4.6	NA		
Hospital de Emergencias de Ate Vitarte (Lima)	5	-12.02584008	-76.91729777	25	Ómicron, Linajes BA.2, BA.4, BA.5, BA.2.12.1	NA		
	6					NA		
	7					NA		
Hospital de Huaycán (Lima)	8	-12.01544188	-76.82024846	19	Ómicron, Linajes BA.1, BA.1.1, BA.2, BA.4, BA.5, BA.2.12.1	NA		
	9					NA		
Hospital Regional de Cusco (Cusco)	10	-13.52354454	-71.95475727	32	Ómicron, Linajes BA.2, BA.4, BA.5, BA.5.2, BA.2.12.1, BA.4.1, BA.5.1, BA.5.1.1, BA.5.2.1, BE.1, BA.4.6, BA.5.6, BA.5.6.1	Ómicron	BA.2	21L
	11					Ómicron	BA.2	21L
Clínica Americana Juliaca (Puno)	12	-15.49743882	-70.13242485	35	Ómicron, Linajes BA.5, BA.5.1, BA.5.2.1, BA.5.2, BA.5.6	Ómicron	BA.5.5	22B
	13					Ómicron	BA.5.1	22B
Hospital Regional de Cajamarca (Cajamarca)	14	-7.183099124	-78.48777246	34	Ómicron, Linajes BA.4, BA.5, BA.4.1, BA.5.1, BA.5.2.1, BA.5.2, BA.4.6, BA.5.1.3, BA.5.6, BA.5.1.3, BA.5.6.1	Ómicron	BA.1.1	21K
	15					NA		
Hospital Sergio Bernales (Lima)	16	-11.91352635	-77.03915675	10	Ómicron, Linajes BA.1, BA.1.1, BA.2	NA		
	17					NA		
Hospital de Tarapoto (San Martín)	18	-6.473262643	-6.473262643	34	Ómicron, Linajes BA.4.1, BA.5.1, BA.5.1.1, BA.5.2.1, BA.5.6.1	NA		
	19					NA		
	20					NA		

NA: no asignado

* Asignación según Instituto Nacional de Salud del Perú para el lugar y semana de muestreo. No fue posible recuperar los datos de Puno durante la semana 24 (16/06/2022), por lo que los datos mostrados son de julio de 2022

Cuando comparamos los resultados obtenidos de las aguas residuales hospitalarias con las muestras de pacientes monitoreados por el INS durante el mismo período, encontramos una similar presencia tanto en la variante donde todas pertenecían a Ómicron, la misma variante circulante en la población peruana, como a los linajes. Solo BA.5.5, no fue reportada por el INS para el área y el momento del muestreo en la región Puno (Tabla 1).

Todas las 14 secuencias, excepto una (WW20) descartada por el software Nextclade debido a su baja calidad, fueron analizadas por las herramientas de Illumina y parecían pertenecer al clado 19A de Ómicron. Sin embargo, este resultado no podía considerarse robusto (las otras herramientas los habían descartado), pero de alguna manera también mostraba la relación subyacente con la variante ómicron.

## DISCUSIÓN

La pandemia de COVID-19 en Perú se caracterizó por un mayor número de casos, muertes por millón de habitantes y exceso total de muertes en comparación con otros países de América del Sur ^19^. Se aducen varias causas para explicar estas cifras, pero principalmente podrían deberse a una estrategia de Salud Pública sin líderes y descoordinada, así como a la falta de infraestructuras ^20^^,^^21^. Paradójicamente, Perú fue el primer país de América Latina en imponer un confinamiento estricto en 2020, logrando una rápida mejoría de la primera ola de casos en junio de 2020 ^22^. Sin embargo, la relajación de las restricciones en agosto de 2020 se asoció con una segunda ola a finales de ese año ^21^. El nuevo pico de casos entre diciembre de 2020 y febrero de 2021 volvió a obligar a medidas de restricción y cuarentenas selectivas en las zonas con mayor incidencia de casos. Un año después, el Ministerio de Salud peruano anunció la aparición de una tercera ola, iniciada así en enero de 2022 y extendida hasta abril de 2022 ^23^^,^^24^. Este estudio surgió en el contexto de esta tercera ola y tuvo como objetivo detectar las variantes del SARS-CoV-2 a partir de diferentes efluentes hospitalarios en el Perú durante el periodo comprendido entre marzo y septiembre del 2022 y abarcó establecimientos de salud de la costa, selva y sierra, mostrando material genético encontrado en las muestras analizadas de los genotipos, lo que indicaría que para las fechas del estudio estarían circulando los linajes mencionados.

Se han detectado variantes del SARS-CoV-2 a partir de muestras respiratorias en Perú, algunas de ellas variantes de interés (VOI) y otras variantes de preocupación (VOC), según la clasificación de los CDC y la OMS en función de su impacto en la salud pública ^25^^,^^26^. Así, en la segunda ola de la pandemia en el Perú se encontraban circulando los linajes Lambda y Gamma. Lambda se enfocó en la región de la costa y sierra, mientras que Gamma en la región de la selva (Loreto) ^11^^,^^27^. De hecho, la variante Lambda (C.37) se detectó por primera vez a nivel mundial en el Perú en agosto de 2020. Por otro lado, la tercera ola estuvo dominada por linajes descendientes de Ómicron BA.1 (B.1.1.529), esta variante fue la única encontrada en nuestro estudio. Además, los linajes descendientes encontrados en las muestras de aguas residuales coincidieron con alguno de los detectados por el INS en pacientes de la misma área geográfica y semana de muestreo. Como se ha visto anteriormente en otros estudios, la detección del SARS-CoV-2 en las aguas residuales puede reflejar la propagación de este virus en la comunidad ^28^^,^^29^. Además, este enfoque permite observar la transmisión no detectada de variantes del SARS-CoV-2 ^30^. En ese sentido, hemos encontrado un linaje de Ómicron, BA.5.5 que no fue reportado por el INS para los lugares y periodo de muestreo, pero que podrían haber estado circulando.

La principal limitación de nuestro estudio fue la calidad de las muestras, lo que nos obligó a descartar un número considerable de muestras. Las secuencias de baja calidad, además de la dificultad para estimar la abundancia relativa del linaje en muestras complejas, no son infrecuentes cuando se lleva a cabo la vigilancia de aguas residuales ^31^. La baja prevalencia de diferentes linajes en esas muestras se señala como la razón principal ^32^, lo que probablemente también explique nuestros resultados. Asimismo, el tiempo de muestreo no coincidió con los grandes picos de casos reportados durante la tercera ola y donde se detectaron muchos linajes en la población.

La fortaleza del estudio radica en que es uno de los primeros estudios en reportar variantes del SARS-CoV-2 en aguas residuales de hospitales en Perú. La vigilancia genómica basada en la secuenciación nos proporciona esta importante información para conocer más sobre la transmisión continua del virus hacia la comunidad. Este estudio, tuvo la participación de diferentes investigadores que forman parte de la red de resistencia antimicrobiana y que han aportado su experiencia y conocimiento para lograr los objetivos planteados; siendo necesario el apoyo de diferentes actores e instituciones. A pesar de las limitaciones, nuestro estudio es otro ejemplo de cómo el análisis de secuenciación de aguas residuales podría ser útil como contraparte para la vigilancia viral en pacientes, especialmente en países donde las pruebas clínicas son aún un desafío.

En conclusión, se evidencia la presencia de variantes del SARSCoV-2 en muestras de aguas residuales hospitalarias y que fueron similares a las reportadas por el sistema de vigilancia en pacientes durante las mismas semanas y áreas geográficas en Perú. El monitoreo de aguas residuales contribuye a proporcionar información sobre la variación ambiental y temporal de virus como el SARS-CoV-2.
